# The Omega-3 Index Response to an 8 Week Randomized Intervention Containing Three Fatty Fish Meals Per Week Is Influenced by Adiposity in Overweight to Obese Women

**DOI:** 10.3389/fnut.2022.810003

**Published:** 2022-02-04

**Authors:** Christine E. Richardson, Sridevi Krishnan, Ira J. Gray, Nancy L. Keim, John W. Newman

**Affiliations:** ^1^Department of Nutrition, University of California, Davis, Davis, CA, United States; ^2^West Coast Metabolomics Center, Genome Center, University of California, Davis, Davis, CA, United States; ^3^United States Department of Agriculture, Agricultural Research Service (USDA-ARS) Western Human Nutrition Research Center, Davis, CA, United States

**Keywords:** dietary intervention, omega-3 fatty acids, Dietary Guidelines for Americans, omega-3 index, omega-3 response, overweight women, fish, typical American diet

## Abstract

**Background:**

The Dietary Guidelines for Americans (DGA) recommends consuming ~225 g/wk of a variety of seafood providing >1.75 g/wk of long-chain omega-3 fatty acids to reduce cardiovascular disease risk, however individual responses to treatment vary.

**Objective:**

This study had three main objectives. First, to determine if a DGA-conforming diet (DGAD), in comparison to a typical American diet (TAD), can increase the omega-3 index (OM3I), i.e., the red blood cell mol% of eicosapentaenoic acid (EPA) + docosahexaenoic acid (DHA). Second, to identify factors explaining variability in the OM3I response to dietary treatment. Third to identify factors associated with the baseline OM3I.

**Design:**

This is a secondary analysis of a randomized, double-blind 8 wk dietary intervention of overweight/obese women fed an 8d rotating TAD (*n* = 20) or DGAD (*n* = 22) registered at www.clinicaltrials.gov as NCT02298725. The DGAD-group consumed 240 g/wk of Atlantic farmed salmon and albacore tuna in three meals with an estimated EPA + DHA of 3.7 ± 0.6 g/wk. The TAD-group consumed ~160 g/wk of farmed white shrimp and a seafood salad containing imitation crab in three meal with an estimated EPA + DHA of 0.45 ± 0.05 g/wk. Habitual diet was determined at baseline, and body composition was determined at 0 and 8wks. Red blood cell fatty acids were measured at 0, 2 and 8 wk.

**Results:**

At 8 wk, the TAD-group OM3I was unchanged (5.90 ± 1.35–5.80 ± 0.76%), while the DGAD-group OM3I increased (5.63 ± 1.27–7.33 ± 1.36%; *p* < 0.001). In the DGAD-group 9 of 22 participants achieved an OM3I >8%. Together, body composition and the baseline OM3I explained 83% of the response to treatment variability. Baseline OM3I (5.8 ± 1.3%; *n* = 42) was negatively correlated to the android fat mass (*p* = 0.0007) and positively correlated to the FFQ estimated habitual (EPA+DHA) when expressed as a ratio to total dietary fat (*p* = 0.006).

**Conclusions:**

An 8 wk TAD did not change the OM3I of ~6%, while a DGAD with 240 g/wk of salmon and albacore tuna increased the OM3I. Body fat distribution and basal omega-3 status are primary factors influencing the OM3I response to dietary intake in overweight/obese women.

## Introduction

Cardiovascular disease (CVD) is a leading cause of mortality, accounting for nearly 18 million deaths worldwide in 2016 [https://www.who.int/news-room/fact-sheets/detail/cardiovascular-diseases-(cvds)]. The consumption of the long-chain omega-3 polyunsaturated fatty acids eicosapentaenoic acid (EPA) and docosahexaenoic acid (DHA) for CVD risk reduction has received much attention (1, 2). Doses as low as 0.7 g/day have been reported to lower triglycerides in subjects with normal fasting triglyceride levels (3). Moreover, systematic reviews of the existent body of evidence support a dose response in CVD risk reduction, with EPA+DHA levels of between 0.25 and 0.5 g/d being sufficient for primary prevention (4, 5). These omega-3 fatty acid levels can be obtained through the consumption of one to three seafood containing meals per week, habits expected to reduce CVD risk, especially when seafood replaces less healthy foods (6). Therefore, dietary modulation of omega-3 fatty acid status by adherence to a healthful diet with high intermittent doses of foods rich in these lipids provides a strategy for CVD risk reduction in the general population. However, species specific and seasonal variability in the n3-PUFA content of foods are important factors influencing consumer exposure (7). Moreover, variability in individual responses to defined n3-PUFA intake are routinely observed and have been attributed to various factors including the basal omega-3 status, body weight, gender, age, and genetics (2, 3, 8–13).

The Dietary Guidelines for Americans (DGA) are evidence-based recommendations that aim to minimize the population risk of developing metabolic diseases including CVD by focusing on the consumption of a healthy, nutrient-dense diet. However, to date these recommendations have not been specifically tested with respect to fish intake and omega-3 status in the context of the entirety of the dietary recommendations. The DGA recommendations are updated every 5 y, and have promoted the weekly consumption 8oz of fatty fish since 2000. The 2010 DGAs used to plan the current intervention specifically recommended the consumption of a minimum of 8oz (i.e., 225 g) per week of seafood providing a minimum daily average of 250 mg of EPA and DHA in a diet high in fruits, vegetables, whole grains, low-fat dairy products, with lower sodium, solid fats and added sugars (14). Dietary polyunsaturated fatty acids (PUFAs) are readily incorporated into cellular phospholipids, and their incorporation into red blood cells (RBCs) combined with the average RBC life span of ~115 days provides an accessible and time-integrated measure of dietary PUFA intake (15, 16).

The omega-3 index (OM3I), a metric expressing the RBC EPA + DHA content as a mole percent of total fatty acids, is a validated diet-sensitive biomarker of omega-3 fatty status (12, 17). The OM3I is reported to be inversely correlated to cardiovascular risk and inflammatory status (18), and directly associated with healthy RBC morphology (19). Therapeutic OM3I cut points of <4, 4–8, and >8 have been established to indicate high, moderate and low CVD risk, respectively (12, 20). The current report describes a secondary analysis of an 8 wk dietary intervention in overweight to obese pre-and post-menopausal women to probe the OM3I response to diets of different quality. To our knowledge, this is the first study to specifically compare the OM3I responses to a DGA-conforming diet (DGAD) to that of a typical American diet (TAD). In the DGAD-group, despite equivalent intake, the 8 wk change in OM3I [ΔOM3I_(Wk0−8)_] in the DGAD ranged from no change to a large change, while the TAD-group showed both increases and decreases in the OM3I. Moreover, android adiposity and body composition were a significant modifier of the ΔOM3I_(Wk0−8)_ response to the DGAD, but not to the TAD intervention.

## Subjects and Methods

### Study Design

The present study used a randomized, double-blind, 8-week dietary intervention in overweight and obese pre-and postmenopausal female. The study is registered at www.clinicaltrials.gov as NCT02298725 and was conducted between Dec 2014 and Mar 2017. All procedures were approved by the University of California, Davis Institutional Review Board. Detailed descriptions of the study design are published (21, 22). Briefly, volunteers were stratified by menopausal status (pre- or post-) and fasting insulin resistance before random assignment. Diets were designed to maintain body weight as eight-day cyclical menus with similar appearance between interventions (21, 22). The TAD was based on the 50th percentile of the National Health and Nutrition Examination Survey (i.e., NHANES) “What We Eat in America” dietary survey and the DGAD was based on food-group recommendations in the 2010 DGAs. Menu planning to achieve differences in omega-3 fatty acid were performed using fatty acid estimates of commercial food products contained in the 2014 version of the Nutrition Data System for Research from the University of Minnesota—Nutrition Coordinating Center (http://www.ncc.umn.edu/products/). In the 2200 calorie DGAD, 80 g portions of baked farmed Atlantic salmon on menu days 1 and 5 were anticipated to provide 1.7 g of EPA + DHA, while a seafood salad, containing 80 g of canned albacore tuna on menu day 7, was anticipated to provide 0.7 g of EPA + DHA. In the 2,200 calorie TAD, 30 g portions of shrimp on menu days 1 and 5, and 100 g portion of a mixed seafood salad on menu day 7 were anticipated to provide 0.5 g of EPA + DHA. The foods providing the majority of omega-3 fatty acids are described in detail in Supplementary Table 1. Accounting for individual caloric prescriptions, the DGAD-group EPA + DHA exposure was 1.7 ± 0.3, 1.8 ± 0.3, and 0.71 ± 0.11 g/d on menu days 1, 5, and 7, with a weekly average of 0.53 ± 0.08 g/d overall. Similarly, in the TAD-group EPA + DHA exposure was 0.10 ± 0.01, 0.10 ± 0.01 and 0.31 ± 0.03 g/d on menu days 1, 5 and 7, with a weekly average of 0.064 ± 0.007 g/d. Individual level exposure estimates are provided in Supplementary Table 2.

### Subjects

Pre- and postmenopausal females (*n* = 44) aged 20–65 years with a BMI (kg/m^2^) of 25–39.9 were recruited for this intervention. Sample size was based on the study primary outcome investigating the impact of diet quality on glucose homeostasis in women with at least one risk factor for the metabolic syndrome (22). Weight stable, sedentary women or those with low physical activity levels and the presence of one or more clinical measures of moderately perturbed glucose homeostasis or lipid metabolism were recruited as previously reported (22). Detailed inclusion and exclusion criteria are provided as Supplementary Information. Any reported omega-3 supplement use was discontinued prior to and during the study.

### Food Frequency Questionnaires

To evaluate the impact of habitual diet and supplement use on the OM3I_(Wk0)_, dietary intake estimates were assessed using Block Food Frequency questionnaires (FFQs). Each participant completed a FFQ ~1–2 weeks prior to the initiation of the intervention, and the responses were used to reflect habitual dietary intake.

### Body Composition

To allow for the analysis of body composition impacts on basal OM3I and the OM3I response to dietary treatment, dual-energy x-ray absorptiometry (DXA) were performed. Once during study week 0 and once during week 8, a whole-body DXA scan was performed using a Hologic® Discovery™ QDR® Series 84994 (Hologic, Inc.). This scan provided values for total lean mass, total fat mass, percent body fat, and estimates of gynoid and android fat distribution.

### Blood Collection and Clinical Chemistry

Fasting blood samples were collected by a licensed phlebotomist, directly into EDTA containing vacutainers, mixed gently, and centrifuged at 1,300 rcf for 10 min at 4°C within 30 min of blood collection. After plasma and buffy coat removal, RBCs from a 5 mL blood tube were suspended in 14 mL phosphate-buffered saline, gently mixed, and pelleted by centrifugation as described above. The washing procedure was repeated and the resulting washed RBCs were transferred to polypropylene Eppendorf tubes and stored at −70°C until analysis.

### Fatty Acid Analyses

Fatty acids methyl esters (FAMEs) were measured in 50 mg aliquots of phosphate-buffered saline-washed red blood cells (RBCs) and daily meal composites by gas chromatography-mass spectrometry in our laboratory. FAMEs were quantified against authentic standards correcting for methodological recoveries of ~5 μmol of d31-tripalmitoylglyceride (Avanti Polar Lipids, Alabaster AL) (23, 24). Data quality assurance/quality control measures included sample randomization, the inclusion of procedural blanks, duplication of 5% of samples, concurrent analysis of laboratory reference materials in each analytical batch, along with the use of isotopically labeled extraction surrogates and derivatization controls. Results were corrected for d31-tripalmitoylglyceride recoveries which were 47 ± 15 and 49 ± 12% for meal composites and RBCs, respectively, with derivatization efficiencies of ~50% as assessed by the presence of 10Z pentadecenote methyl ester. Replicate precision of RBCs and meal composites were ~15% for FAMEs above the detection limit.

### Statistics

All statistical analyses were performed in JMP Pro v 15.1 (SAS Institute, Cary, NC). Data normality was assessed using Shapiro-Wilk tests and Q-Q plots of raw, log and Johnson transformed data. Optimized Johnson transformations were generated by the employed software program. Statistical tests associated with specific questions are described in the following subsections.

### Baseline Characteristics and Dietary Fatty Acids

Group mean differences in baseline parameters and meal composite fatty acids were compared with Student's *t*-test (α = 0.05). To investigate parameters associated with the baseline OM3I [OM3_(Wk0)_], physiological and habitual diet parameters were evaluated alone and together using linear models.

### Intervention Diet Impact on RBC Fatty Acids and the OM3I

Linear mixed models were used to determine if intervention diets differentially changed the OM3I and/or other RBC fatty acids. Participant was assigned as a random effect, diet-group, week and diet-group by week interactions were fixed effects. If main or interaction effects were identified, Least Square Means Tukey HSD were used for adjusted multiple comparison tests (α = 0.05). K means clustering of the OM3I 8wk rate of change [i.e., the slope of the regression of the OM3I_(Wk0)_ to OM3I_(Wk8)_ vs wk] and the average of the OM3I_(Wk0)_ and OM3I_(Wk2)_ were used to segregate participants by their OM3I rate of change in each diet group.

### Determinants of OM3I Change

Regression analyses were used to evaluate the associations between the 8 wk change in the OM3I [ΔOM3I_(Wk8−0)_], OM3I_(Wk0)_, diet, body composition and age. To determine if unique subgroups existed within each diet group with respect to the influence of the OM3I_(Wk0)_ on ΔOM3I_(Wk8−0)_, the ΔOM3I_(Wk0−8)_:OM3I_(Wk0)_ ratio was analyzed using a hierarchical cluster analyses with a Ward agglomeration. Stepwise linear regressions using the Bayesian Information Criterion (BIC) as the stopping function were used to build models of the ΔOM3I_(Wk8−0)._ Multiple comparisons errors were controlled by false discovery rate p-value adjustments.

### Predictors of OM3I_(Wk0)_

Body composition and FFQ estimated dietary factors were explored as predictors of OM3I_Wk0_ using stepwise linear regressions with the BIC stopping function. Collinearity of OM3I_(Wk0)_, habitual diet and body composition factors were assessed by clustering using an implementation of the VARCLUS algorithm, and key variables were visualized using Pearson's correlation heat maps.

## Results

### Participant Enrollment, Completion, and Characteristics

**Figure 1:**
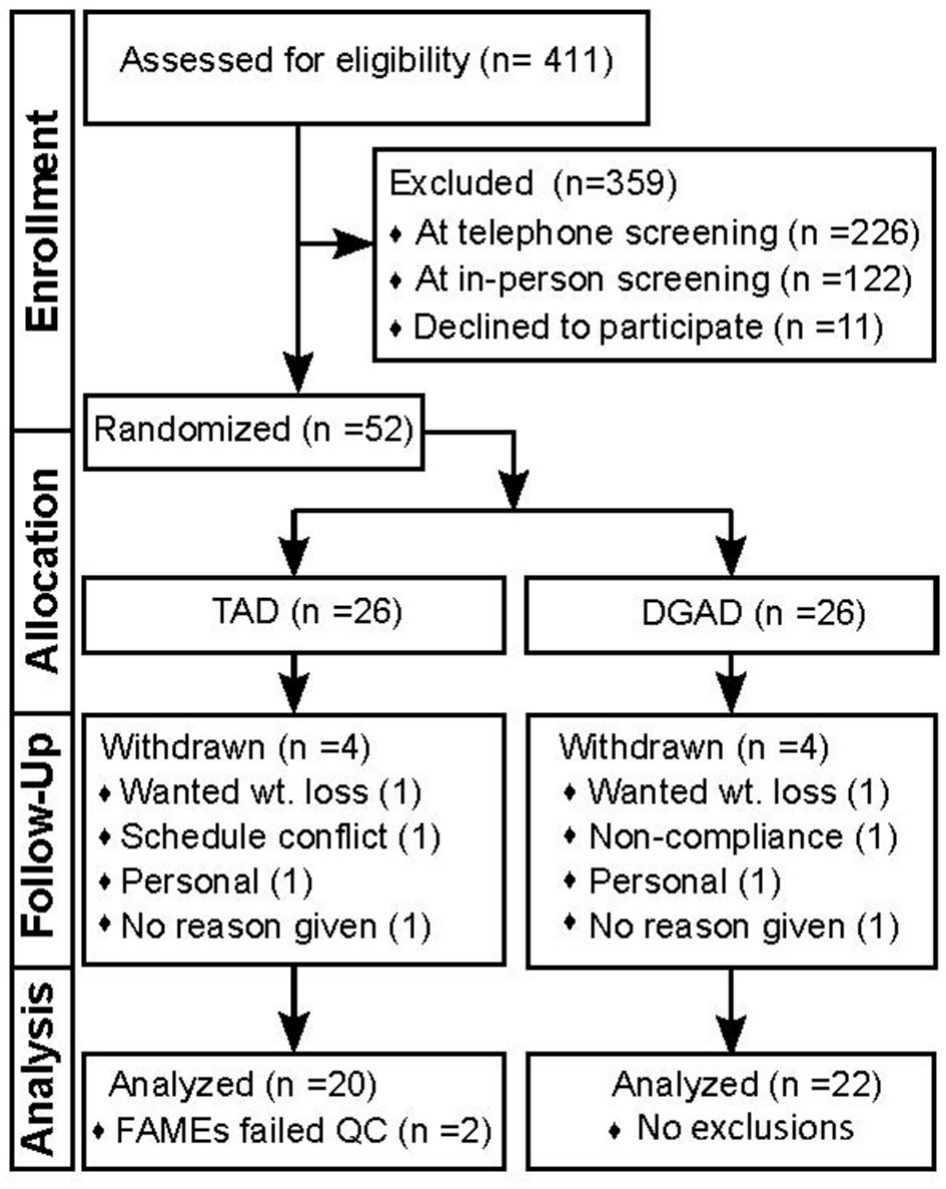
Study flow diagram documenting participant enrollment, allocation, follow-up and analysis. This diagram conforms the Consolidated Standards of Reporting Trials (i.e., CONSORT). TAD, typical American diet; DGAD, Dietary Guidelines for American's diet; FAMEs, red blood cell fatty acid analysis; QC, quality control criterion.

A complete diagram of the enrollment, allocation, follow-up and analysis is provided in Figure 1. Of the 52 participants enrolled, eight withdrew and 44 completed the intervention. No adverse effects were reported. Results were missing for one baseline and one terminal RBC fatty acid measurement from two distinct individuals in the TAD group due to poor analytical performance. Therefore, analysis for the primary and secondary outcomes in this study were performed with *n* = 22 for the DGAD and *n* = 20 for the TAD groups. Summarized baseline characteristics for participants analyzed for the primary outcome by group are shown in Table 1. Individual data are provided in Supplementary Table 3.

**Table 1:** Study cohort baseline clinical and body composition characteristics^a^.

**Variables**	**Units**	**TAD**	**DGAD**	***p***
*N*		20	22	
Age	y	48.0 ± 10.0	50.7 ± 12.6	0.4
OM3I	mol%	5.90 ± 1.35	5.63 ± 1.27	0.3
Fasting TGs	mg/dL	111 ± 49	143 ± 101	0.6
HOMA-IR		3.77 ± 2.75	3.22 ± 2.43	0.5
BMI	kg/m^2^	33.0 ± 3.68	32.0 ± 4.0	0.5
Body weight	kg	88.5 ± 13.8	88.6 ± 15.6	0.9
Total mass^b^	kg	87.0 ± 13.7	86.9 ± 15.3	0.9
Lean mass^b^	kg	47.1 ± 7.1	47.0 ± 8.0	0.9
Fat mass^b^	kg	38.0 ± 7.7	37.8 ± 7.8	1
Android fat mass^b^	kg	3.33 ± 0.99	3.48 ± 1.04	0.6
Gynoid fat mass^b^	kg	6.36 ± 1.45	6.37 ± 1.62	1
Android: gynoid^b^	%	52.1 ± 0.11	56.0 ± 15.1	0.4
% Body fat^b^	%	43.2 ± 3.9	43.3 ± 3.0	0.9
% Trunk fat^b^	%	44.2 ± 5.3	45.3 ± 3.7	0.6

a *All values are means ± SD. Groups mean differences were not detected by Student's t-test (α = 0.05). DXA, dual-energy x-ray absorptiometry; DGAD, Dietary Guidelines for Americans diet; OM3I, omega-3 index; TAD, typical American diet; TG, triglycerides*.

b *Reported values were derived from DXA measurements*.

### Dietary Fatty Acid Composition

The average fatty acid composition of both diet plans are shown in Table 2. These analyses confirmed that the TAD contained higher saturated fatty acids (SFA) and monounsaturated fatty acids (MUFA) and lower omega-3 polyunsaturated fatty acids (n3-PUFAs) compared to the DGAD. Relative to the TAD, the DGAD was significantly enriched in DHA, as well as alpha-linolenic acid (ALA), with EPA approaching significance (*p* = 0.1). The relative abundance of n6-PUFAs was equivalent between the two diets, and both meal plans contained a substantial amount of ALA.

**Table 2:** Average meal composite fatty acid composition (mol%)^a^.

**Fatty acid**	**TAD**	**DGAD**	**DGAD fold TAD**	***p***
C10:0	1.7 ± 0.7	0.90 ± 0.40	−1.9	0.007
C12:0	3.7 ± 2.1	0.63 ± 0.43	−5.9	<0.0001
C14:0	5.6 ± 1.7	2.8 ± 1.0	−2.0	0.0005
C15:0	0.39 ± 0.1	0.22 ± 0.07	−1.8	0.0021
C16:0	24 ± 3	19 ± 4	−1.3	0.0034
C17:0	0.32 ± 0.07	0.18 ± 0.06	−1.8	0.0014
C18:0	8.5 ± 0.9	6.7 ± 1.0	−1.3	0.0033
C19:0	0.025 ± 0.004	0.023 ± 0.005	—	0.3
C20:0	0.097 ± 0.029	0.11 ± 0.02	—	0.3
C21:0	0.0035 ± 0.0008	0.0035 ± 0.0007	—	0.9
C22:0	0.099 ± 0.082	0.13 ± 0.06	—	0.3
C24:0	0.046 ± 0.028	0.064 ± 0.024	0.4	0.1
C14:1n5	0.43 ± 0.09	0.18 ± 0.09	−2.4	0.0001
C16:1n7	1.3 ± 0.5	0.90 ± 0.4	−1.4	0.053
C18:1n9	31 ± 4	39 ± 4	0.3	0.0015
C18:1n7	1.6 ± 0.3	2.0 ± 0.4	0.3	0.021
C19:1n9	0.0088 ± 0.0049	0.0020 ± 0.003	−4.4	0.002
C20:1n9	0.072 ± 0.030	0.11 ± 0.07	—	0.3
C18:2n6 (LA)	18 ± 5	22 ± 4	—	0.09
C18:3n6	0.024 ± 0.011	0.025 ± 0.02	—	0.8
9c,11t-CLA	0.085 ± 0.017	0.036 ± 0.02	−2.4	<0.0001
C20:3n6	0.018 ± 0.0054	0.017 ± 0.009	—	0.5
C20:4n6 (AA)	0.087 ± 0.036	0.093 ± 0.049	—	0.8
C22:4n6	0.019 ± 0.0083	0.020 ± 0.010	—	0.7
C22:5n6 (n6-DPA)	0.0061 ± 0.0042	0.013 ± 0.010	—	0.3
C18:3n3 (ALA)	2.0 ± 0.6	3.6 ± 1.2	0.8	0.0065
C18:4n3	0.014 ± 0.027	0.05 ± 0.07	—	0.6
C20:3n3	0.0028 ± 0.0017	0.0076 ± 0.0059	1.7	0.047
C20:4n3	0.0017 ± 0.0007	0.0080 ± 0.0100	—	0.6
C20:5n3 (EPA)	0.0056 ± 0.0083	0.11 ± 0.17	18.6	0.1
C22:5n3	0.045 ± 0.012	0.086 ± 0.080	—	1.0
C22:6n3 (DHA)	0.021 ± 0.006	0.41 ± 0.73	18.5	0.036
SFA	45 ± 7	30 ± 6	−1.5	0.0005
MUFA	35 ± 4	43 ± 4	0.2	0.0021
n-6 PUFA	18 ± 5	23 ± 4	0.3	0.1
n-3 PUFA	2 ± 1	4 ± 2	1.0	0.0041

a *All values are the means ± SD of menu day 1thru 8 composite results. Group differences evaluated by Student's t-tests (α = 0.05)*.

*DGAD, Dietary Guidelines for Americans diet; TAD, typical American diet; AA, arachidonic acid; n6-DPA, docosapentaenoic acid; ALA, alpha-linolenic acid; EPA, eicosapentaenoic acid; DHA, docosahexaenoic acid; LA, linoleic acid*.

### Intervention Diet Impact on the OM3I and Other RBC Fatty Acids

The DGAD increased RBC omega-3 PUFAs resulting in an increase in the OM3I, with marginal reductions in omega-6 PUFAs. No changes in SFAs or MUFAs were observed. As seen in Table 3, the DGAD-group showed increases in the RBC EPA and DHA mol% (*p* < 0.0001), and a modest decreases in C22:5n6 (n6-docosapentaenoic acid; n6-DPA; *p* = 0.03) was suggested. Significant changes in other RBC fatty acids were not detected, however linoleic acid concentrations in the DGAD group tended to decline over time differentially between groups (p_Wk_ = 0.1; p_DietxWk_ = 0.1). The level of ALA in RBCs was near the analytical detection limit in the assay, and the resulting data were not available for analysis. This is in agreement with reports of RBC ALA content of ~0.1 mol% (25).

**Table 3:** RBC fatty acid mole percent composition^a^.

	**TAD (*****n*** **=** **20)**			**DGAD (*****n*** **=** **22)**			***p***		
**Fatty acid**	**Wk 0**	**Wk 2**	**Wk 8**	**Wk 0**	**Wk 2**	**Wk 8**	**Diet**	**Wk**	**Diet × Wk**
C14:0	1.28 ± 0.73	1.05 ± 1.00	1.55 ± 1.20	1.29 ± 0.93	1.11 ± 0.49	0.993 ± 0.660	0.7	0.3	0.4
C15:0	0.387 ± 0.300	0.371 ± 0.074	0.288 ± 0.540	0.346 ± 0.130	0.319 ± 0.082	0.306 ± 0.390	0.7	0.5	0.8
C16:0	29.5 ± 3.8	30.1 ± 3.3	29.8 ± 1.0	28.7 ± 4.1	30.0 ± 3.1	29.7 ± 3.3	0.5	0.4	0.8
C17:0	0.401 ± 0.11	0.362 ± 0.12	0.449 ± 0.340	0.430 ± 0.110	0.356 ± 0.095	0.404 ± 0.150	0.4	0.1	0.4
C18:0	24.2 ± 4.2	23.6 ± 4.1	23.7 ± 6.3	26.0 ± 5.4	24.9 ± 4.6	24.3 ± 5.2	0.2	0.5	0.8
C18:1n9	10.9 ± 1.3	10.7 ± 1.1	10.6 ± 5.5	10.8 ± 1.7	10.7 ± 1.6	10.9 ± 1.7	0.3	0.8	0.8
C18:1n7	2.07 ± 0.38	2.03 ± 0.26	1.92 ± 0.23	1.95 ± 0.65	2.02 ± 0.44	1.98 ± 0.43	0.9	0.9	0.5
C18:2n6 (LA)	6.52 ± 0.94	6.42 ± 0.90	6.36 ± 6.20	6.31 ± 0.92	5.94 ± 0.73	5.91 ± 0.95	0.8	0.1	0.1
C20:3n6	1.25 ± 0.24	1.28 ± 0.35	1.33 ± 1.60	1.39 ± 0.46	1.42 ± 0.43	1.37 ± 0.19	0.3	1.0	0.4
C20:4n6 (AA)	9.55 ± 1.40	9.93 ± 1.50	10.1 ± 2.2	9.21 ± 1.30	9.29 ± 1.40	9.22 ± 1.40	0.4	0.4	0.5
C22:4n6	4.06 ± 0.730	4.11 ± 0.83	4.28 ± 0.26	4.22 ± 0.88	4.20 ± 0.85	4.00 ± 0.88	0.4	0.9	0.2
C22:5n6 (n6-DPA)	0.834 ± 0.320	0.877 ± 0.280	0.841 ± 0.230	0.899 ± 0.270^ab^	0.945 ± 0.260^a^	0.727 ± 0.300^b^	0.5	0.03	0.1
C20:5n3 (EPA)	0.430 ± 0.130^AB^	0.381 ± 0.082^ABC^	0.345 ± 0.640^C^	0.383 ± 0.15^BC^	0.422 ± 0.13^BC^	0.493 ± 0.18^A^	0.3	0.4	<0.0001
C22:5n3	3.14 ± 0.73	3.20 ± 0.84	3.02 ± 1.20	2.90 ± 0.75	2.93 ± 0.59	2.89 ± 0.82	0.4	0.7	0.8
C22:6n3 (DHA)	5.47 ± 1.10^B^	5.57 ± 0.69^B^	5.45 ± 0.46^B^	5.24 ± 0.89^B^	5.47 ± 1.30^B^	6.86 ± 1.30^A^	0.5	<0.0001	<0.0001
Σ SFA	55.8 ± 3.5	55.5 ± 3.6	55.8 ± 9.5	56.7 ± 4.1	56.7 ± 4.1	55.7 ± 3.9	0.4	0.8	0.5
Σ MUFA	12.9 ± 1.6	12.7 ± 1.3	12.5 ± 1.6	12.7 ± 2.2	12.7 ± 2.0	12.8 ± 2.0	0.7	0.9	0.7
Σn-6 PUFA	22.2 ± 2.3	22.6 ± 2.6	22.9 ± 1.2	22.0 ± 2.6	21.8 ± 2.2	21.2 ± 2.6	0.8	0.9	0.2
Σn-3 PUFA	9.04 ± 1.40^AB^	9.15 ± 0.79^AB^	8.82 ± 1.8^B^	8.52 ± 1.30^B^	8.83 ± 1.80^B^	10.3 ± 1.6^A^	0.2	0.01	0.0003
OM3I	5.90 ± 1.35^B^	5.95 ± 1.2^B^	5.8 ± 0.72^B^	5.63 ± 1.27^B^	5.89 ± 0.95^B^	7.36 ± 1.40^A^	0.4	<0.0001	<0.0001

a *All values are means ± SD. Treatment effects on RBC fatty acids were tested using least squares regression models incorporating diet, time, and diet x time interactions controlling for participant as a random effect with Tukey HSD post-hoc tests. For each fatty acid residue, values within diet groups with different superscripts are different at α = 0.05. If significant diet × time interactions were detected, changes are annotated with uppercase letters. TAD, typical American diet; DGAD, Dietary Guidelines for Americans diet; OM3I, omega-3 index; AA, arachidonic acid; n6-DPA, docosapentaenoic acid; ALA, alpha-linolenic acid, EPA, eicosapentaenoic acid; DHA, docosahexaenoic acid; LA, linoleic acid*.

**Figure 2:**
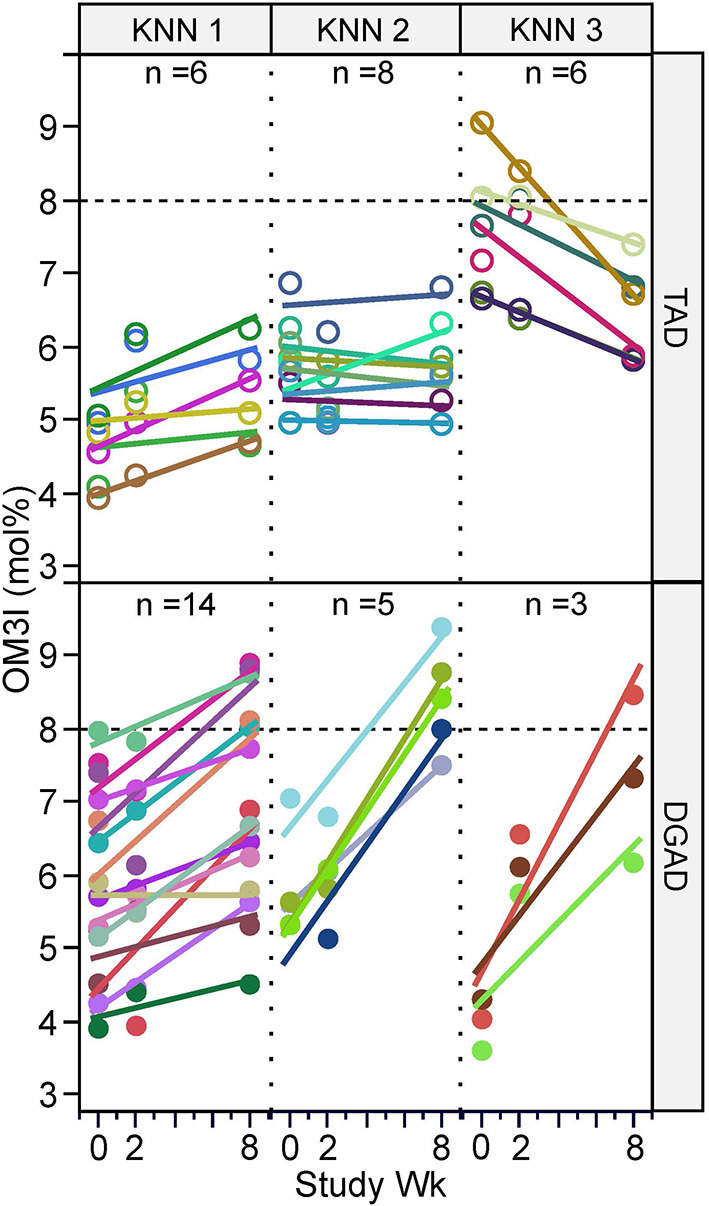
Omega-3 index response rate variance by diet group and time. Participants within the typical American diet (TAD; *n* = 20) and Dietary Guidelines for Americans diet (DGAD; *n* = 22) groups were clustered based on the 0–8 wk rate of OM3I change and the average OM3I_(Wk0)_ and OM3I_(Wk2)_, by K-means clustering, with each diet group being segregated into three groups of participants with different responses. As a significant change between Wk0 and Wk2 was not detected (Table 3), this term was applied to provide a better estimate of the baseline OM3I for this analysis and particularly stabilized the TAD-group clustering. Participant measures and regression lines share common colors.

While the OM3I_(Wk0)_ of the TAD (5.90 ± 0.13) and DGAD (5.63 ± 1.27) did not differ by group (Table 1), the diets differentially affected the OM3I over time (*p* < 0.0001; Table 3). In the DGAD-group, despite equivalent calorie adjusted dietary intake the ΔOM3I_(Wk0−8)_ in the DGAD ranged from −0.1 to 4.5% points. In the TAD-group, the ΔOM3I_(Wk0−8)_ ranged from −2.3 to 1.2% points. On average, the DGAD OM3I_(8Wk)_ was increased to 7.36 ± 1.40, while the TAD-group mean OM3I_(8Wk)_ was unchanged. To further characterize the range of responses observed in each diet group, the rate of change in the OM3I between 0 and 8 wk and the log of the fold-change between Wk0 and Wk2 were analyzed by K-means clustering. As a significant change between Wk0 and Wk2 was not detected (Table 3), this 2 week average was applied to provide a better estimate of the baseline OM3I for this analysis. This approach particularly stabilized the TAD-group clustering with little effect on that of the DGAD-group. This analysis identified three unique clusters in each diet group (Figure 2). In the TAD-group, the average OM3I increased by 0.8 ± 0.3 (*n* = 6) in cluster 1, remained unchanged (i.e., −0.1 ± 0.3, *n* =8) in cluster 2, and decreased by 1.4 ± 0.6 (*n* = 6) in cluster 3. In the DGAD-group, the OM3I increased 1.0 ± 0.5 (*n* = 14), 2.6 ± 0.5 (*n* = 5), and 3.4 ± 0.8 (*n* = 3) in clusters 1, 2, and 3, respectively. In the DGAD-group, the OM3I increased in 21 of 22 (i.e., ~95%) participants, as opposed to only seven of 20 (i.e., 35%) in the TAD-group.

### Determinants of OM3I Change

**Figure 3:**
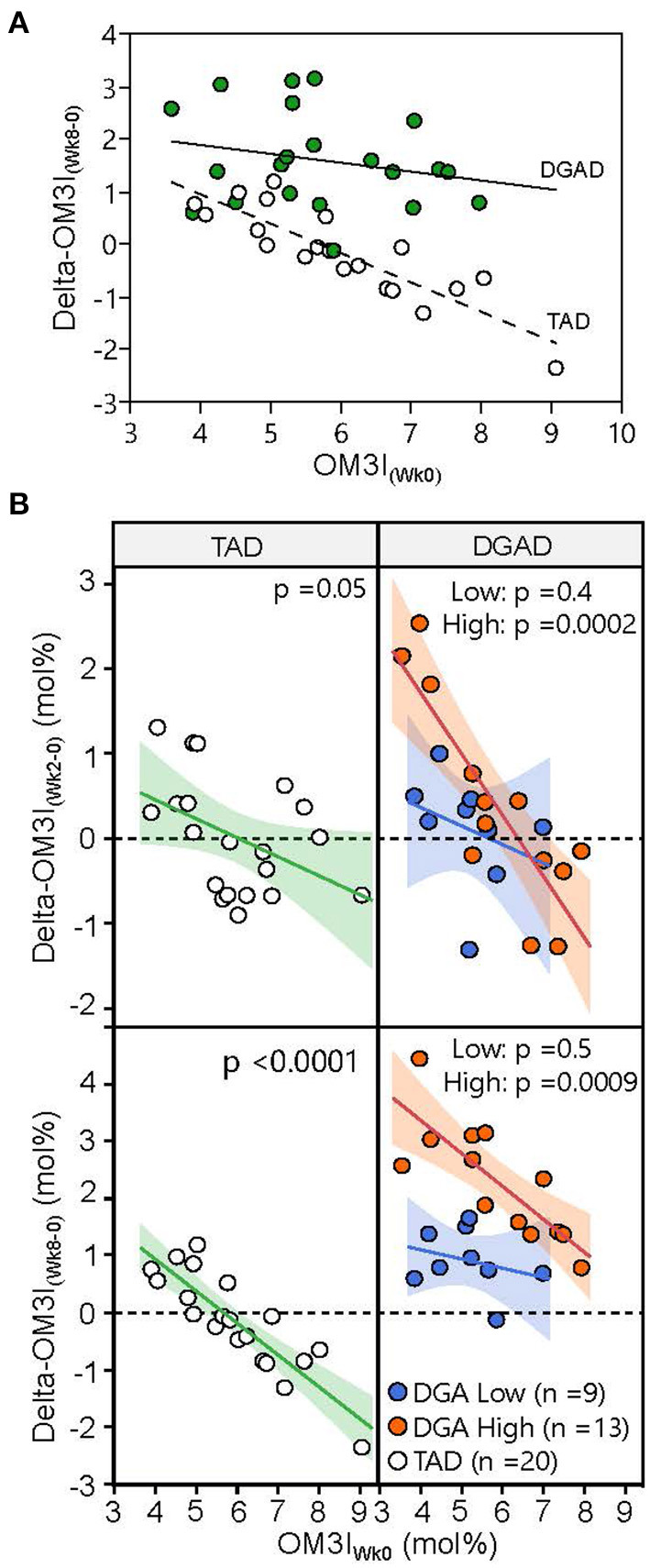
Variance in omega-3 index response to typical American diet (TAD) and Dietary Guidelines for American's diet (DGAD) interventions. **(A)** The 8 wk OM3I change (OM3I_(Wk8−Wk0)_) expressed as a function of baseline omega 3 index (OM3I_(Wk0)_). Diet (*p* < 0.0001), OM3I_(Wk0)_ (*p* = 0.0003), and diet x OM3I_(Wk0)_ interactions (*p* = 0.036) were detected. **(B)** Participants within diet groups were clustered according to their OM3I_(Wk8−0)_:OM3I_(Wk0)_ ratio by hierarchical cluster analysis and projected onto both 2 wk (top) and 8 wk (bottom) data. Hierarchical cluster analysis identified low (blue) and high (orange) responding subgroups in the DGAD group. The correlations between OM3I_(Wk0)_ and magnitude of change were significant in the DGAD high, but not low response subgroups.

When analyzing the diet groups together (*n* = 42), the ΔOM3I_(Wk0−8)_ showed significant associations with OM3I_(Wk0)_ (*p* < 0.0001), diet-group (*p* < 0.0001) and OM3I_(Wk0)_ x diet-group interactions (*p* = 0.0001), with the baseline OM3I alone explaining 34% of the OM3I response (Figure 3A). However, as n3-PUFA exposure differed by intervention, an analysis by diet-group was performed. In the TAD-group 74% of the variance in the ΔOM3I_(Wk0−8)_ was explained by negative correlations with the OM3I_(Wk0)_ (*p* < 0.0001). However, in the DGAD-group, only 31% of the ΔOM3I_(Wk0−8)_ variance was explained by the OM3I_(Wk0)_ (*r*^2^ = 0.31, *p* = 0.007). To determine if sub-groups existed within diet groups with different relationships between the ΔOM3I_(Wk0−8)_ and the OM3I_(Wk0)_, an exploratory hierarchical cluster analysis of the ΔOM3I_(Wk8−0):_OM3I_(Wk0)_ ratio was performed on each diet group. While a single group was observed in the TAD-group, the DGAD-group was split into high (*n* = 13) and low (*n* = 9) response clusters (Figure 3B). The OM3I_(Wk0)_ and ΔOM3I_(Wk8−0)_ were negatively correlated in the high response group (*p* < 0.009), but not in the low response group (*p* = 0.5).

Next, we sought to determine if combining dietary and body composition measures with OM3I_(Wk0)_ could better explain the response to treatment in the DGAD-group. Models including OM3I_(Wk0)_ and the FFQ estimated habitual EPA + DHA daily intake increased the explained variance by 25% (*r*^2^ = 0.56, RMSE = 0.16, *p* = 0.0004). However, the ΔOM3I_(Wk8−0)_ showed a counterintuitive negative correlation with intake [i.e., when adjusting for OM3I_(Wk0)_, as dose increased the ΔOM3I_(Wk8−0)_ decreased]. We then introduced DXA-based body composition, BMI and age using a BIC-limited stepwise linear regression, which yielded a model accounting for 70% of the variance in the DGAD ΔOM3I_(Wk8−0)_. This false discovery rate adjusted least squares regression model (*r*^2^ = 0.70, RMSE = 0.12; *p* = 0.002) included negative associations with the OM3I_(Wk0)_ (p_adj_ = 0.027), trunk % fat (p_adj_ = 0.012), lean body mass (p_adj_ = 0.013) and positive associations with android fat mass (p_adj_ = 0.027) and BMI (p_adj_ = 0.033). Of these factors, android fat mass was positively correlated with lean body mass, BMI and trunk % fat (*p* < 0.001), along with the average FFQ estimated EPA+DHA daily intake. Notably, multidimensional outliers in highly correlated independent variables were not observed.

**Figure 4:**
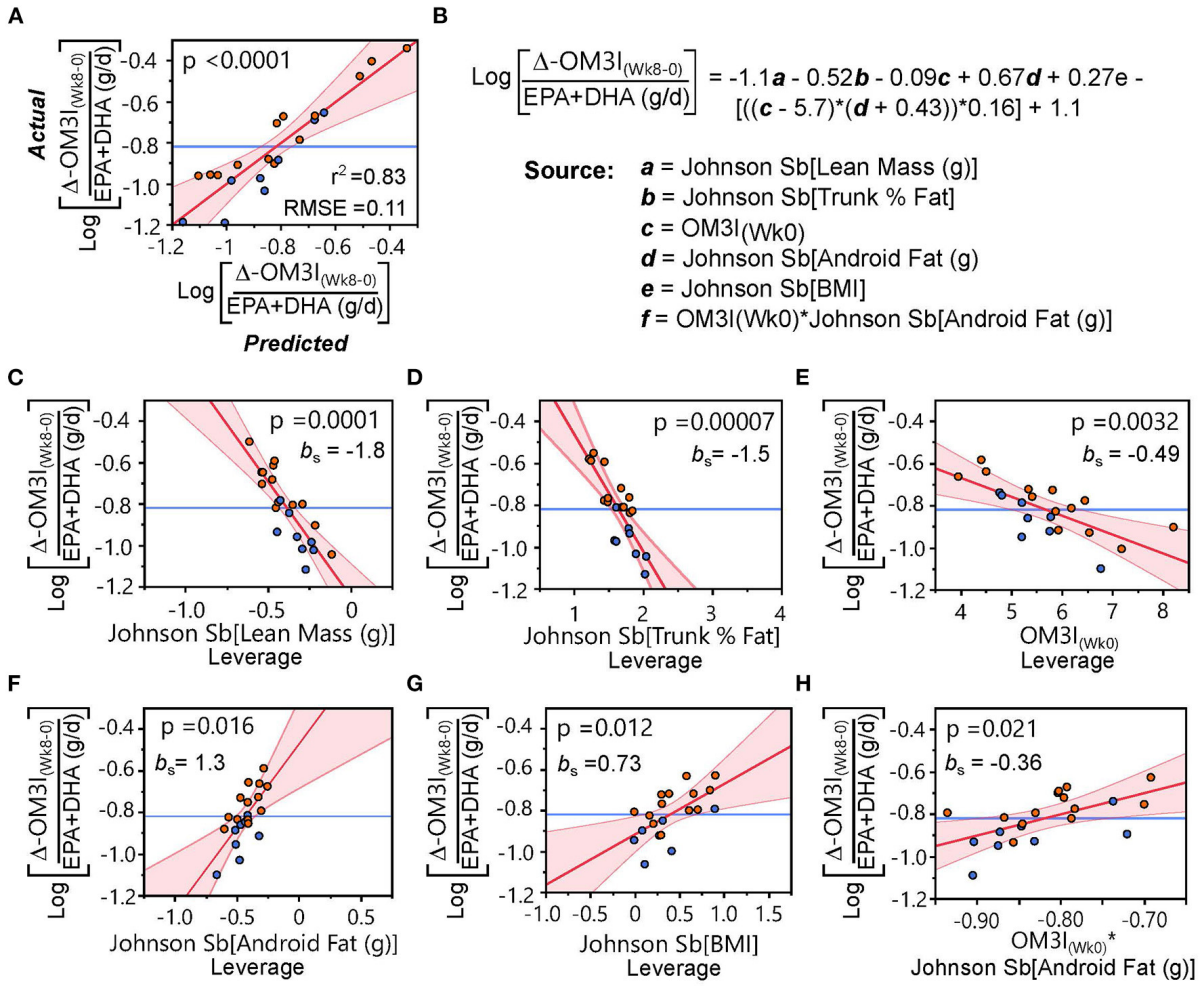
Body composition and the baseline omega-3 index (OM3I_(Wk0)_) explain the dose-dependent 8 wk change in the OM3I (Log[ΔOM3I_(Wk8−0)_/(EPA+DHA)]). **(A)** Actual x Predicted plot from the stepwise linear regression of the Log[ΔOM3I_(Wk8−0)_/(EPA+DHA)] using body composition factors and OM3I_(Wk0)_ explaining 83% of the variance. **(B)** Regression equation and model components. **(C–H)** Leverage plots with p-values and the standardized beta coefficient (*b*_s_) for each model component: **(C)** lean body mass; **(D)** trunk %fat; **(E)** OM3I_(Wk0)_; **(F)** android fat; **(G)** BMI; **(H)** OM3I_(Wk0)_ × android fat interactions. Symbols: orange circles—high responding subgroup of the Dietary Guidelines for American Diet group defined in Figure 3B; blue circles—low responding subgroup of the Dietary Guidelines for American Diet group defined in Figure 3B. *b*_s_, standardized beta-coefficient; RMSE, root mean square error.

Exploring model factor interactions revealed OM3I_(Wk0)_ interactions with android fat mass (*p* = 0.014), lean mass (*p* = 0.020), and BMI (*p* = 0.025). Modeling the intervention dose dependent change [i.e., [ΔOM3I_(Wk8−0)_/[EPA+DHA] (g/d)] using the body composition and OM3I_(Wk0)_ together yielded the strongest model (*r*^2^ = 0.83; *p* < 0.0001, RMSE = 0.011; Figure 4). The rank order of factor impact in this model was lean mass >trunk %fat >android fat >BMI >OM3I_(Wk0)_ > OM3I_(Wk0)_ x android fat interactions. These analyses suggest that after adjusting for the OM3I_(Wk0)_, the ΔOM3I_(Wk8−0)_ response to the daily dose of EPA + DHA is lower as body size increases, but higher as android fat deposition increases.

### Predictors of the OM3I_(Wk0)_

**Figure 5:**
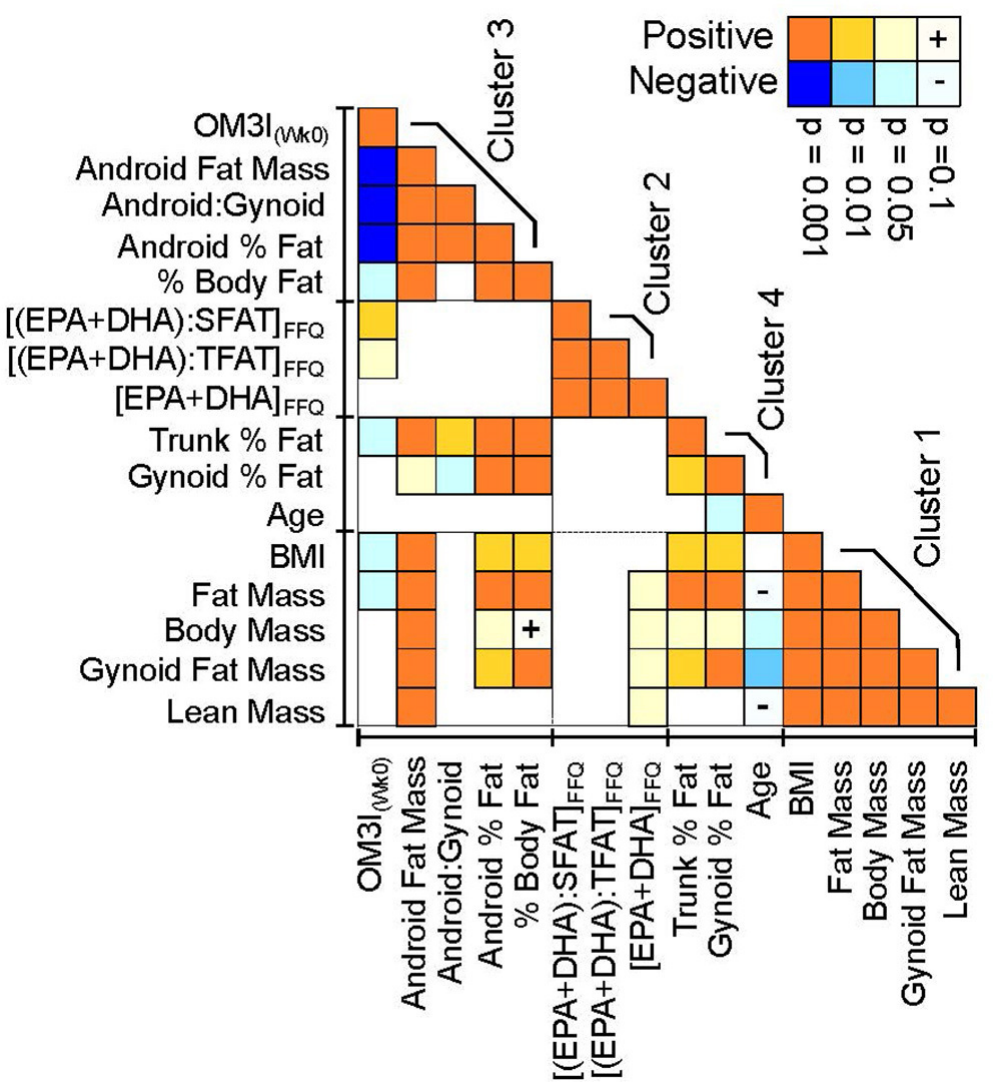
Pearson's correlation heatmap of the baseline omega-3 index, habitual omega-3 fatty acid factors and DXA-dependent body composition measures. Correlation statistics are calculated on normally transformed data, with positive (orange) and negative (blue) correlation strength indicated by color intensity. Variables were clustered in JMP v16 using implementation of the SAS VARCLUS algorithm. Clusters are ranked by decreasing order of explained variance. Variables are ordered within clusters by their correlation strength with the baseline omega-3 index (OM3I_(Wk0)_). FFQ, food frequency questionnaire; SFAT, dietary saturated fat; TFAT, dietary total fat.

As the OM3I_(Wk0)_ has a strong influence on ΔOM3I_(Wk8−0)_, FFQ estimated habitual dietary intake data (Supplementary Table 4) and Wk0 body composition (Supplementary Table 3) were explored for factors associated with the OM3I_(Wk0)_ using all subjects with complete baseline data (*n* = 43). As seen in Figure 5, the OM3I_(Wk0)_, habitual omega-3 fatty acid intake and body composition variables condense into four correlated variable clusters. The OM3I_(Wk0)_ showed strong negative correlations with the android fat mass, android:gynoid fat ratio and the android % fat (*p* < 0.001). The OM3I_(Wk0)_ was also positively correlated with the habitual (EPA+DHA): habitual saturated fat (SFAT) ratio, the habitual (EPA+DHA): habitual total dietary fat (TFAT) ratio, and the habitual MUFA: habitual TFAT ratio (data not shown). Of these, the habitual (EPA+DHA):SFAT ratio and habitual (EPA+DHA):TFAT ratio were the most strongly correlated with the OM3I_(Wk0)_ (*p* < 0.01). The FFQ estimated EPA + DHA intake alone was not correlated with the OM3I_(Wk0)_. Exclusion of a single FFQ data set outlier resulted in a significant correlation between the OM3I_(Wk0)_ and EPA + DHA intake (*p* < 0.05), however the ratios remained more strongly associated (*p* < 0.001).

Combining habitual diet and body composition factors into a stepwise linear regression analysis explained 43% of the variance in the OM3I_(Wk0)_ (*r*^2^ = 0.43, *p* < 0.0001), in a model which included android fat mass (*p* = 0.0001) and the habitual (EPA+DHA):SFAT ratio (*p* = 0.0026) or the habitual (EPA+DHA):TFAT ratio (*p* = 0.0031; Supplementary Figure 1). The android fat mass was not correlated (*p* = 0.9) with either habitual (EPA+DHA):SFAT or habitual (EPA+DHA):TFAT.

## Discussion

Since 2000, the DGAs have recommended the weekly consumption of ~225 g of seafood per week to obtain at least 1.7 g of dietary EPA + DHA (https://www.dietaryguidelines.gov/). The present study was established to determine if consuming a diet based on the 2010 DGA for 8 wk could beneficially alter the OM3I as compared to a TAD, in overweight to obese pre-and postmenopausal women. Secondary analyses were performed to identify factors associated with variability in the ΔOM3I_(Wk8−0)_ and influencing the OM3I_(Wk0)_, an important determinant of the OM3I response.

As expected, 8 weeks of the DGAD increased the average OM3I relative to the TAD. The TAD provided only 0.45 ± 0.05 g/wk of EPA+DHA from three meal containing 160 g/wk of farmed white shrimp and a seafood salad containing imitation crab. While this represents ~70% of the recommended intake of seafood, these meals contained ~30% of the recommended daily dose of EPA+DHA. Consuming this diet for 8 weeks did not change the group average OM3I. However, with this low EPA+DHA intake, individuals with OM3I_(Wk0)_ <5% tended to increase, while those >7% tended to decrease. Therefore, dietary long chain n3-PUFAs representing the average consumption in the United States were only sufficient to achieve and/or maintain an OM3I in a moderate CVD risk range. In contrast, the experimental DGAD provided an estimated 3.7 ± 0.6 g/wk (i.e., 0.53 ± 0.08 g/d) of EPA+DHA from 240 g/wk of salmon and albacore tune in three meals of the 8d rotating menu, with the variance due to differences in individual caloric prescriptions. These meals essentially met the level of recommended seafood intake, but contained ~2.3x the minimum 1.75 g/wk of EPA + DHA recommendation. This level of EPA +DHA intake was slightly above 3.5 g/wk (i.e., 0.5 g/d) recommended by the Academy of Nutrition and Dietetics to provide primary protection for CVD risk (26). DGAD consumption increased the OM3I from 5.6 ± 1%, by 1.7 ± 1.1% points to 7.4 ± 1.35%. This average change is in good agreement with the 2.4% point change obtained with 0.6 g/d EPA+DHA provided as fish oil supplement (9). However, among the 22 individuals, responses ranged from 0 to 4.5% points. Specifically, 14 of 22 individuals (~63%) on the DGAD showed OM3I improvements of ~1% point, seven had 2.3–4.5% point changes and one person was unchanged. In all, nine DGAD-group participants exceeded the lower limit of the OM3I CVD low risk cut point of 8% OM3I by the end of the study. Therefore, it is possible to move individuals from a moderate to low CVD risk category based on the OM3I in 8 weeks by adhering to DGAD recommendations with significant EPA and DHA sources in only three meals of fatty fish per week. However, substantial interindividual variability in the ΔOM3I_(Wk8−0)_ is observed and larger doses or durations are likely required for much of the population.

Factors influencing the OM3I response to supplementation include the n3-PUFA dose, the OM3I_(Wk0)_, body mass, age, sex, and physical activity (8, 9, 13). In the current study, when considering all subjects magnitudes of improvement slowed as the OM3I_(Wk0)_ increased, consistent with previous findings (8, 11, 13). However, correlations between the OM3I_(Wk0)_ and the ΔOM3I_(Wk8−0)_ were much weaker in the DGAD vs. the TAD groups. In the TAD-group, the OM3I_(Wk0)_ explained ~70% the ΔOM3I_(Wk8−0)_, as opposed to only ~30% in the DGAD-group. A subgroup analysis of the DGAD-group suggested that the OM3I_(Wk0)_ alone was a poor predictor of the ΔOM3I_(Wk8−0)_ in 9 of the 22 DGAD-participants, and body composition was identified as a major driver of the unexplained variance. In particular, after adjusting for the OM3I_(Wk0)_, the ΔOM3I_(Wk8−0)_ was negatively influenced by the percent trunk fat and lean body mass, but positively influenced by the android fat mass and BMI. Therefore, it appears that n3-PUFA intervention efficacy was enhanced in women with greater abdominal and overall adiposity after adjusting for OM3I_(Wk0)_ and body size. Importantly, these effects remained after considering the mass specific dose of EPA+DHA. Moreover, while high doses of n3-PUFAs may influence body weight and fat mass in obese women (27) these factors were unchanged in the current population. To the best of our knowledge, the influence of adiposity on the OM3I response treatment has not been reported. Why women with greater adiposity would respond more strongly to the same dose of dietary EPA+DHA is unclear. Regardless, elevations in android fat deposition are reportedly associated with elevated plasma triglycerides, endothelial dysfunction, type 2 diabetes, and fatty liver disease (28–31), all conditions beneficially affected by high omega-3 fatty acid intake.

Factors reported to influence the OM3I include both the habitual diet and body composition. For instance in large cross-sectional studies, FFQ-based estimates of n3-PUFA intake correlate with the OM3I, and an increase in weekly additional servings of fish has been associated with a 6–13% increase in the OM3I (32–35). In the current study, the FFQ estimated habitual EPA + DHA intake of individual participants was poorly correlated with the OM3I_(Wk0)._ However, expressing these data as a percentage of the total dietary fat, or its relative abundance with the saturated fat intake revealed modest positive correlations. Exploring associations between body composition and the OM3I_(Wk0)_, android fat mass was found to have a strong negative association with the OM3I_(Wk0)_, which was independent of the dietary factors. Similarly, a recent study in >3000 middle aged to elderly Chinese found that RBC DHA, but not EPA was negatively associated adiposity indices, the strongest of which being the android fat deposition (36). Contradicting this finding, in a previous study of older women, a high dietary omega-3/omega-6 ratio was associated with higher android fat (37). That study also found that a high dietary SFA/PUFA ratio was positively associated with android fat, a relationship that was weakly observed in the current data (*p* = 0.1).

## Limitations

Various factors may have influenced the actual individual exposure to omega-3 fatty acids in the diet. While foods were purchased in lots to minimize variance in nutritional exposures, due to seasonal variability in fish stocks and farmed salmon feed, the true omega-3 exposure may vary from the NDSR estimates reported here. Unfortunately, the small sample amounts (i.e. 50 mg) used for meal composite fatty acid analysis led to unacceptable variance in the absolute values of some measures, and were not available for estimating dietary intake. Also, measured fatty acids in meal composites were generated at a single time point during the course of the study and the composition of dietary exposures may differ from the measured composite meals. In addition, we cannot by 100% certain that individuals consumed all provided foods. While dietary compliance was monitored by inspection of returned food containers and surveys of participants regarding the consumption of study and non-study foods and significant deviations were not recorded, errors in these estimates may exist and not be uniformly distributed across the study population, or over the time of the study. However, such variability likely reflects how individuals actually eat and thus the results likely reflect the response to the presented foods. While the DGAD conformed to the recommended intake of >225 g/wk of seafood, having a total of 240 g/wk, the seafood selected contained ~2.3x the minimum recommended EPA+DHA. Therefore, consuming DGADs conforming to the recommended seafood intake but using foods with lower EPA+DHA would be expected to have less robust results. The current study has a relatively small sample size and was conducted in a narrow group of overweight to obese women. Therefore, the findings may not be generalizable to leaner populations or to men. In addition, the presented subgroup analyses should not be considered to represent the frequency of responsiveness in the population. Also, while physiological associations with the OM3I_(Wk0)_ and the ΔOM3I_(Wk8−0)_ are reported that explain a substantial proportion of the observed variance, these associations do not demonstrate a causal relationship and other factors including genetics influencing both long chain omega-3 fatty acid biosynthesis and triglyceridemic responsiveness were not evaluated. Finally, the clinical relevance of the observed changes in the OM3I with respect to CVD risk cannot be evaluated in the current study.

## Conclusion

Adherence to an ALA-rich DGAD with ~4 g/wk of EPA+DHA provided in three meals/wk for 8 wks increases the OM3I in pre- and post-menopausal women with a basal OM3I of 5.6 ± 1.3%, while an ALA-rich TAD providing ~0.5 g/wk of EPA+DHA did not change the OM3I of ~6%, which is in the moderate CVD risk range. After adjusting for the OM3I_(Wk0)_ and body size, the effect of the omega-3 fatty acid rich intervention was higher in women with greater android and overall adiposity. These findings support food recommendations in the Dietary Guidelines seeking to increase an individuals' omega-3 fatty acid status. However, more clarity on omega-3 content of seafood, and or the duration of exposure are warranted. Further, it should be appreciate that an individual's adiposity and a predominance of central fat may positively affect an individuals' response to dietary omega-3 fatty acids.

## Data Availability Statement

The original contributions presented in the study are included in the article/Supplementary Material, further inquiries can be directed to the corresponding author/s.

## Ethics Statement

The studies involving human participants were reviewed and approved by University of California, Davis Institutional Review Board. The patients/participants provided their written informed consent to participate in this study.

## Author Contributions

JN and NK conceived and developed the research plan. JN, SK, IG, and NK conducted the research. JN and IG analyzed the data and performed the statistical analysis. CR and JN wrote the primary manuscript. SK and NK provided significant editorial input of manuscript. All authors have primary responsibility for final content and read and approved the final manuscript.

## Funding

This study was supported by the National Dairy Council, Campbell Soup Co., and USDA-ARS Projects 2032-51530-022-00D and 2032-51530-025-00D. The USDA is an equal opportunity provider and employer.

## Conflict of Interest

The authors declare that the research was conducted in the absence of any commercial or financial relationships that could be construed as a potential conflict of interest.

## Publisher's Note

All claims expressed in this article are solely those of the authors and do not necessarily represent those of their affiliated organizations, or those of the publisher, the editors and the reviewers. Any product that may be evaluated in this article, or claim that may be made by its manufacturer, is not guaranteed or endorsed by the publisher.

## Abbreviations

ALA: alpha-linolenic acid (C18:3n3)
AA: arachidonic acid (C20:4n6)
CVD: cardiovascular disease
EPA: eicosapentaenoic acid (C22:5n3)
ΔOM3I_(Wk8−0)_: 8 wk omega 3 index change from baseline
DGA: Dietary Guidelines for Americans
DGAD: Dietary Guidelines for Americans diet
DHA: docosahexaenoic acid (C22:6n3)
DXA: dual-energy x-ray absorptiometry
FAME: fatty acid methyl esters
FFQs: Block food frequency questionnaires
LA: linoleic acid (C18:2n6)
n6-DPA: n6-docosapentaenoic acid (C22:5n6)
mol%: mole percent composition
OM3I: omega-3 index
OM3I_(Wk0)_: baseline omega 3 index
RBC: red blood cell
SFAT: dietary saturated fat
TAD: typical American diet
TFAT: total dietary fat
TG: triglycerides.
