# Supplementary material for: The Omega-3 Index Response to an 8 Week Randomized Intervention Containing Three Fatty Fish Meals Per Week Is Influenced by Adiposity in Overweight to Obese Women
Source: Front Nutr. 2022 Feb 4;9:810003. doi: 10.3389/fnut.2022.810003 (PMC8855121; doi:10.3389/fnut.2022.810003)
Supplement: Supplementary file 5 [file Data_Sheet_1.PDF]

Supplemental Information:

Subject Inclusion / Exclusion Criteria

Inclusion criteria included the presence of one or more of the following clinical measures of moderately perturbed glucose homeostasis or lipid metabolism:

1) 100-126 mg/dL fasting glucose;
2) 140-199mg/dL oral glucose tolerance test 2h glucose;
3) Quantitative Insulin Sensitivity Check Index score <0.315;
4) homeostasis model assessment of insulin resistance (HOMA-IR) >3.67;
5) HbA1c between 5.7-6.5,
6) fasting triglycerides >150mg/dL;
7) high density lipoprotein cholesterol <50mg/dL.

Exclusion criteria included:

1) moderate or strenuous physical activity >30 min/d on >5 d/wk;
2) resting blood pressure >140/90mmHg; hemoglobin <11.5g/dL;
3) total cholesterol >300mg/dL;
4) low density lipoprotein cholesterol >189mg/dL;
5) triglycerides >400mg/dL;
6) clinically diagnosed abnormal thyroid or liver function;
7) the presence of any metabolic disease, gastrointestinal disorders, cancer or other serious chronic diseases; pregnancy or lactation;
8) use of tobacco;
9) use of medications for elevated lipids or glucose;
10) regular use of prescription or over-the-counter medications in the 6mo before enrolling into the study;
11) weight change of >5% of body weight in the 6mo before enrolling into the study;
12) working overnight shifts or forced regular all-night wake cycles;
13) dietary restrictions interfering with the intervention foods.
