# Supplementary figures and images for: The Omega-3 Index Response to an 8 Week Randomized Intervention Containing Three Fatty Fish Meals Per Week Is Influenced by Adiposity in Overweight to Obese Women
Source: Front Nutr. 2022 Feb 4;9:810003. doi: 10.3389/fnut.2022.810003 (PMC8855121; doi:10.3389/fnut.2022.810003)

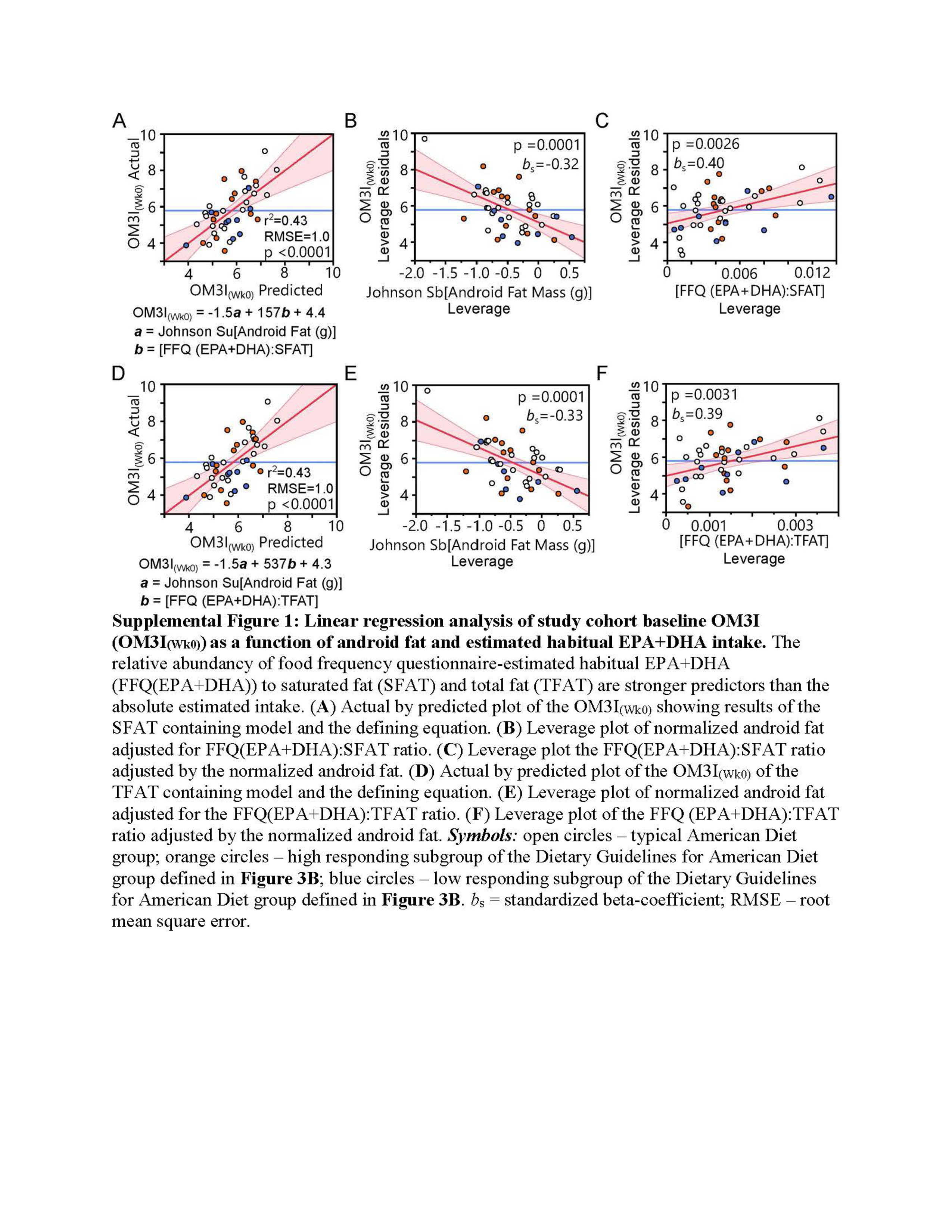

Supplement: Supplementary file 6 [file Image_1.jpg]
